# Massive fungal biodiversity data re-annotation with multi-level clustering

**DOI:** 10.1038/srep06837

**Published:** 2014-10-30

**Authors:** Duong Vu, Szániszló Szöke, Christian Wiwie, Jan Baumbach, Gianluigi Cardinali, Richard Röttger, Vincent Robert

**Affiliations:** 1 Bioinformatics group, CBS-KNAW Fungal Biodiversity Centre, Utrecht, The Netherlands; 2 Computational Systems Biology, Max Planck Institute for Informatics, Saarbrücken, Germany; 3 Institute for Mathematics and Computer Science, University of Southern Denmark, Odense, Denmark; 4 University of Perugia, Perugia, Italy

## Abstract

With the availability of newer and cheaper sequencing methods, genomic data are being generated at an increasingly fast pace. In spite of the high degree of complexity of currently available search routines, the massive number of sequences available virtually prohibits quick and correct identification of large groups of sequences sharing common traits. Hence, there is a need for clustering tools for automatic knowledge extraction enabling the curation of large-scale databases. Current sophisticated approaches on sequence clustering are based on pairwise similarity matrices. This is impractical for databases of hundreds of thousands of sequences as such a similarity matrix alone would exceed the available memory. In this paper, a new approach called MultiLevel Clustering (MLC) is proposed which avoids a majority of sequence comparisons, and therefore, significantly reduces the total runtime for clustering. An implementation of the algorithm allowed clustering of all 344,239 ITS (Internal Transcribed Spacer) fungal sequences from GenBank utilizing only a normal desktop computer within 22 CPU-hours whereas the greedy clustering method took up to 242 CPU-hours.

Biomedical data is being generated at an increasingly fast pace which can most prominently be observed in DNA sequence databases. At the CBS-KNAW Fungal Biodiversity Centre in the Netherlands alone, we collect ~50,000 sequences every year for the classification and identification of fungal species. One routine operation in computational biology is to search for clusters of similar (homologous) genomic sequences in such databases. Despite the high degree of complexity of currently available search routines, the massive number of available sequences makes the quick and correct identification of large groups of similar sequences practically impossible. The problem is more evident for much larger databases like GenBank where approximately six million fungal DNA sequences are currently available for download.

Our initial motivation for this work was the automatic re-annotation of databases of fungal species where many of the sequences have not been edited or checked for years, which may cause data curation problems^1^. When using sequence-based comparison techniques alone, errors can easily propagate through the database. Hence, we nowadays use sophisticated clustering methods for identifying groups of homologous sequences; fungal DNA sequences in our case. The bottleneck with these approaches is the necessity for pairwise-similarity matrix, which assigns each pair of sequence entries in the database a similarity score (the BLAST similarity score^2^, for instance). To give an example, computing such BLAST scores for all 344,239 ITS (Internal Transcribed Spacer) fungal sequences from GenBank took approximately 16 days on a modern desktop computer. For the millions of sequences that will be generated with the next generation of sequencing technology there is no hope of computing such matrices efficiently.

In this work we present the following contributions: (1) We demonstrate that such complete similarity matrices are not essential for detecting clusters of homologous sequences with high accuracy. (2) We quantify and use this effect to show how we may benefit from incomplete similarity information. (3) We introduce a method called Multi-Level Clustering (MLC), which avoids the computation of the majority of the sequence comparisons, and therefore, significantly reduces the total run-time for clustering-based remote homology predicting. In our study, we compared MLC with two sophisticated data-partitioning methods that have proven accurate for BLAST-based sequence clustering: Transitivity Clustering (TransClust)^3^ and a connected component-based clustering (CCBC) method^4^. We also compared MLC with the greedy clustering method used by UCLUST^5^ and CD-HIT^6^ which are considered the most efficient tools to cluster large-scale datasets. For two small manually curated gold standard datasets (medical fungal DNA sequences and protein families) we observe no or only a marginal drop in accuracy when compared MLC with TransClust (TC) and CCBC on complete similarity- matrices. By applying MLC, we were able to cluster all of the 344,239 ITS (Internal Transcribed Spacer) fungal sequences at species level on a single modern desktop computer in 22 hours, which is about 20 times faster than computing a complete similarity matrix and 10 times faster than applying the greedy clustering method. This is a major step forward for the fungal community, since the ITS locus is the officially recommended fungal DNA barcode sequence that underlies fungal biodiversity research worldwide^7^. The availability of the newly curated datasets will significantly support addressing several typical ITS-based barcoding problems, such as automatic taxonomy assignment, discovery of new species, or subspecies. Even though MLC was developed especially for the automatic re-annotation of our fungal database, it is a particularly useful tool in all fields of data partitioning where we deal with similarity functions, such as protein family detection.

## Methods

### Computing of similarity measure

MLC is designed to improve accuracy and speed for pairwise similarity measures from any type of data. However, here we focus on sequence homology^2^,^8^,^9^,^10^,^11^,^12^,^13^. The pairwise alignment function used is our own implementation of BLAST^1^ and can compute a pairwise similarity matrix of 10,000 DNA sequences within 20 minutes on a computer with a single core 64bit CPU and 8 GB RAM. We use Blast percent identity as a similarity measure, since it is wildly used for identification of fungal species. The manual curation of the medial fungal DNA gold standard dataset studied in this paper was done using this similarity measure. In addition, using Blast percent identify, we could also improve the quality of TransClust^3^ on the other gold standard dataset (the protein dataset). In^3^, the authors used Blast E-value as a similarity measure to cluster the protein dataset with TransClust. They could archive the best quality (*F-measure*) of 0.91, while we could archive the best quality of 0.928 for TransClust using Blast percent identity (see Table 1).

### Connected components based clustering algorithm (CCBC)

Several approaches have been suggested for the clustering of sequences^3^,^4^,^14^,^15^,^16^,^17^,^18^,^19^,^20^. Especially when handling large datasets, the method has to be able to find optimal solutions while being very efficient in its use of memory and computational time. The algorithm to search for connected components (CCBC) as equivalence classes in graph theory^21^,^22^ has shown to be very efficient in protein-sequence clustering^4^. It can be computed in linear time using either breadth-first search or depth-first search^21^ if a sequence similarity matrix is given initially. However, when this matrix is not provided, the time complexity of CCBC is the time complexity to compare all the sequences which is O(*N*^2^) with *N* the number of the sequences.

### Representative sequence

In practice, it is almost impossible to provide a sequence similarity matrix when dealing with massive numbers of sequences due to memory and time constraints. To overcome this issue, we aim to show that such similarity matrices are not essential for detecting clusters of homologous sequences with high accuracy. Our idea started from an observation: if two sequences are similar, then there is a high chance that the neighbors of the two sequences are also similar. Thus, it is not necessary to compare these sequences. We could avoid the majority of the sequence comparisons by comparing only *representative sequences* of groups of similar sequences. The *representative sequence* of a group can be any sequence of the group. Ideally, it should be central within the distribution of the sequences belonging to the group. It is shown in^23^ that the *central sequence* that maximizes the similarity measures to the other sequences of the group is the most representative of the distribution of the sequences in the group.

### Greedy clustering algorithm (GC)

The idea of using representative sequences in clustering has been employed by the greedy algorithm which has shown to be very efficient in time and memory for clustering large-scale datasets with UCLUST^5^ and CD-HIT^6^. It is described as follows: (1) sort sequences in order of decreasing length; (2) select the first sequence as the representative sequence of the first group; (3) for each remaining sequence, search for a similar representative with respect to the clustering similarity measure (or *threshold*). If a representative is found, the sequence will be in the same group with the representative. Otherwise, the current sequence will become a new representative; (4) repeat until every sequence has found a group. The greedy algorithm is very fast as its complexity is *O*(*Nm*), where *N* is the number of the sequences and *m* the number of the final groups. However, it does not guarantee a high accuracy for clustering.

### MultiLevel Clustering algorithm with one level (MLC1)

**Figure 1:**
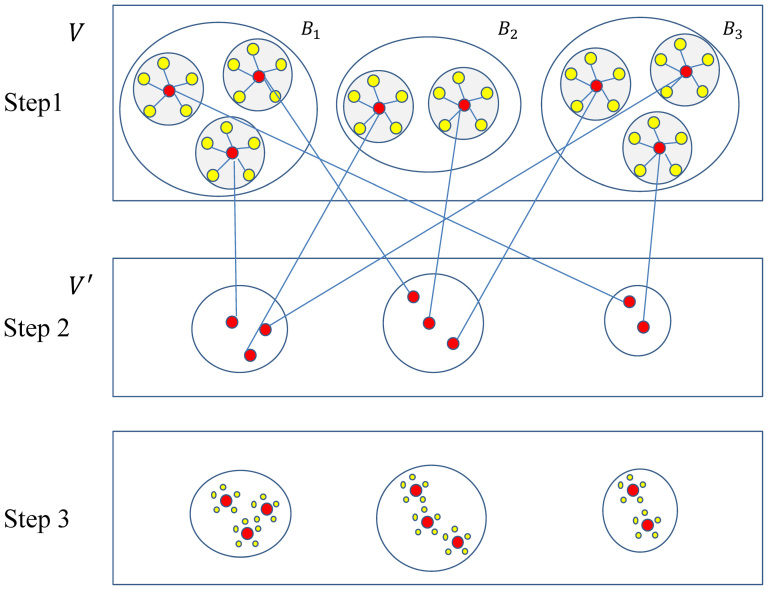
Multilevel clustering with one level (MLC1).

In this section, the MultiLevel Clustering algorithm with one level (MLC1) is proposed to avoid the majority of sequence comparisons while retaining high accuracy. The principle of the method is illustrated in Figure 1.

Suppose that at the beginning the set of sequences is partitioned into three small blocks *B*_1_, *B*_2_ and *B*_3_. Then sequences in each block are clustered as equivalent classes into small groups (Step 1). The representative sequences (in red color) of the obtained small groups are then collected and re-clustered (Step 2). In the end, all equivalent sequences of a representative sequence will be grouped to the same cluster (Step 3). Thus, instead of comparing all sequences with each other, only a reduced number of comparisons have to be computed. Having described the principles on which MLC1 is based, the algorithm is formulated as follows:

**Input:** a set of *N* sequences, a similarity threshold *t*, and the number *K* of blocks or groups to be partitioned.

**Step 1:**

1. Partition sequences into small blocks {*B*_1_, . . . , *B_K_*};
2. For each block *B_i_* with 1 ≤ *i* ≤ K:
  - Cluster sequences in *B_i_* with threshold *t*;
  - Compute the representative sequences for the obtained groups;

**Step 2**: Cluster the representative sequences with threshold *t*;

**Step 3**: Group all equivalent sequences to the cluster of their representative sequence.

Depending on the purpose, one can use different clustering tools such as^3^,^4^,^16^,^18^,^19^,^20^ to cluster sequences in Steps 1.2.a and 2. To archive our aim, we use GC for fast clustering in Step 1.2.a, and CCBC for highly accurate clustering in Step 2.

Unlike GC, the representative sequence of a group in Step 1.2.b of MLC1 is ideally chosen as the central sequence of the group. However, when the number of sequences of a group is big, finding this central sequence can be computationally expensive. To avoid this problem, we heuristically determine the representative of a group as the *almost-central sequence* which is the central sequence of a subset of the group containing *k* sequences, where *k* is a very small number such that the time to compute a similarity matrix of *k* sequences is insignificant. To build this subset, we order the sequences of the group respecting the similarity measure to their representative sequence obtained by Step 1.2.a using GC. Let *s*_1_,...,*s_n_* be the sequences after the ordering, then 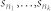 is the subset of *k* sequences where 
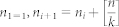
 for 1 ≤ *i* < *k*. It is shown with our experiments that the accuracy of MLC1 depends on the choice of the representative sequence in a group when the number of the sequences in a block is big. The experiments were done on the two reference gold standard datasets when the representative sequence of a group was chosen as the first sequence; the central sequence and the almost-central sequence of the group. Supplementary Fig. 1–2 shows that the best quality (*F-measure*) produced by MLC1drops in the case that the representative sequences were chosen as the first ones of the groups.

The complexity of MLC1 is *O*(*m*(*N* + *m*)), where *m* is the number of the obtained representative sequences. MLC1 is fast when *m* is small. The number *m* can be reduced to minimal if similar sequences are put into the same block initially. This can be archived by pre-clustering the sequences based on prior knowledge of current classification, or using GC with a lower threshold.

### MultiLevel Clustering algorithm (MLC)

**Figure 2:**
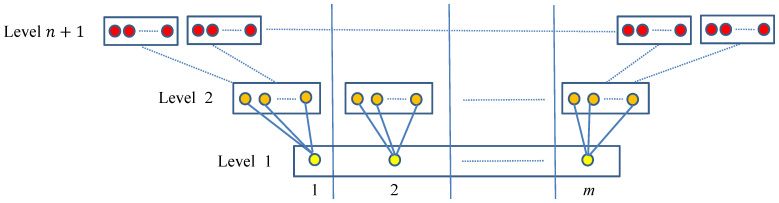
Multilevel clustering (MLC).

As can be seen in the previous sections, it is time and memory-consuming to cluster a large-scale dataset with a high threshold as the number of expected groups is big. In this section, we propose the MultiLevel Clustering algorithm (MLC) to solve this problem. Our idea starts from the observation given in the previous section: it is very fast to cluster a dataset of homologous sequences. Thus, instead of clustering a dataset with the given threshold, it is more efficient to cluster the dataset with a lower threshold. The obtained groups will then be clustered with a slightly higher threshold, and so on till we reach to the given threshold. To guarantee a high accuracy for clustering, MLC1 is used to cluster the obtained groups in each iteration of MLC. MLC is illustrated in Figure 2 and described formally as follows:

**Input:** a set (or group) of *N* sequences and a similarity threshold *t*. Let *t*_1_,...,*t_n_* be a set of thresholds, where 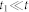, *t_i_* < *t_i_*_+1_ for 1 ≤ *i* < *n*-1, and *t_n_* = *t*Initially, *i* = 1.

**Step 1**: For each group of the current iteration *i*-th, cluster the sequences of the group using MLC1 with threshold *t* if the number of the sequences is small enough (less than a given number *s*); otherwise cluster the sequences with threshold *t_i_* and the obtained groups will be reconsidered for the next iteration.

**Step 2:** *i* = *i* + 1;

**Step 3:** Go to Step 1 until *i*>*n*.

### Recursive MultiLevel Clustering algorithm (rMLC)

It is shown by experiments that MLC is as accurate as CCBC, and is faster than GC. Thus, to speed up the whole process of clustering, the *recursive* Multilevel Clustering (rMLC) algorithm is proposed. The principle of rMLC is the same as of MLC. However, instead of using GC in Step 1 and CCBC in Step 2 of MLC1, rMLC is applied recursively if the number of the sequences in these steps is bigger than a given number *M*; otherwise MLC is applied.

### Run-time performance of clustering

In order to evaluate the run-time performance of a clustering algorithm, let *f* be a value computed as the fraction of the number of sequence comparisons needed for that clustering algorithm divided with the number of sequence comparisons needed to compute a pairwise-similarity matrix. The smaller the value of *f*, the smaller the cost of the clustering algorithm is. For CCBC and other clustering algorithms that require a similarity matrix, this *f-value* is 1.

### Quality of clustering

A common way to evaluate the quality of a clustering is to compare the clustering result against some *reference standard* dataset. A *reference standard* dataset consists of pre-clustered sequences containing well-known and curated information, created, updated and checked by experts. The *F-measure* function introduced in^18^ and used in^3^ will be applied to evaluate the clustering algorithms presented in this paper.

Given a set V of sequences, let *C* = (*C*_1_, . . . , *C_l_*) be the *reference standard* partition of *V*, and *K* = (*K*_1_ *, . . . , K_m_*) the partition obtained by clustering homologous sequences on V . The *F*-measure function *F* (*K,C*) is defined as follows: 
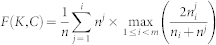
where *n* is the number of sequences in *V*, *n_i_* is the number of sequences in *K_i_*, *n^j^* is the number of sequences in *C_j_*, and 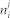 is the number of sequences in 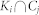 for 1 ≤ *i* ≤ *m* and 1 ≤ *j* ≤ *l*.

The value of *F (K, C)* is between 0 and 1. When *F*-measure is equal to 1, the clustering method reproduces the gold standard perfectly, whereas is does not work at all when F equals 0.

### Optimal threshold

The *optimal threshold* to cluster a reference standard dataset is the one that gives the highest *F*-measure value for clustering. Different clustering algorithms can have different optimal thresholds on the same reference dataset. The clustering tools CCBC, TC and GC always produce a fixed value for the optimal threshold on a given dataset. For MLC1 and MLC, this optimal threshold can be changed depending on their input such as the number of sequences in each block of MLC1, the input set of thresholds for MLC, and the initial ordering of the sequences.

## Implementation and evaluation

MLC1, MLC and rMLC are simple to implement and it can also easily be cloud enabled in order to deal with large-scale datasets. The tested version of MLC together with the pairwise alignment and clustering algorithms were implemented in C++ and Visual Basic.Net. To evaluate the efficiency of MLC1 and MLC, CCBC, GC and TC^3^,^4^ were also implemented and installed. Two reference standard datasets were used and analyzed on a single computer with 64bit CPU and 8 GB RAM. In the following sections, results obtained by the CCBC, GC, MLC1, MLC and TC tools on these reference datasets are compared and discussed. It is noted that since the sizes of the two reference datasets are small, rMLC were not applied.

### Datasets

The first reference dataset consists of 866 protein sequences belonging to 91 families of the Amidohydrolases superfamily which was published by Brown *et al.* in^24^ and has been used as a reference dataset to evaluate approaches on sequence clustering including TC^3^.

The second standard dataset consists of 2800 reference ITS (Internal Transcribed Spacer) sequences of 421 species from the ISHAM-ITS reference database (http://its.mycologylab.org). This dataset was built for the identification of medical fungal species which were manually curated and analyzed by experts.

The sequences of these two datasets were sorted in order of decreasing lengths initially.

### Run-time performance and quality of MLC1

In this section, we study the best input setting for MLC1. The comparison of MLC1 with other tools will be given in the next section. The run-time performance (*f*-value) and quality (*F*-measure) of MLC1 with a given threshold on two reference datasets were studied with and without pre-clustering step, and with the number of sequences in each block of Step 1 increasing. When the number of sequences in each block is 0, MLC1 behaves the same as CCBC, and therefore, *f* = 1. It is noted that the cost of the pre-clustering step was also taken into account when computing these *f*-values in our experiments. To compute the almost-central sequence for a group in Step 1.2.b of MLC1, *k* = 10 was fixed.

The thresholds used in this study should reflect the curated classification. Thus, for the Amidohydrolases protein dataset, the threshold 0.3379 was chosen as it was the optimal threshold of MLC1 on this dataset when the sequences were ordered by GC using the threshold 0.05, and the number of sequences in each block was given as 50. For the medical fungal ITS reference sequences, the threshold 0.9811 was used. It was the optimal threshold of MLC1 when the sequences were order by GC using the threshold 0.5 and the number of the sequences in each block was 100.

**Figure 3:**
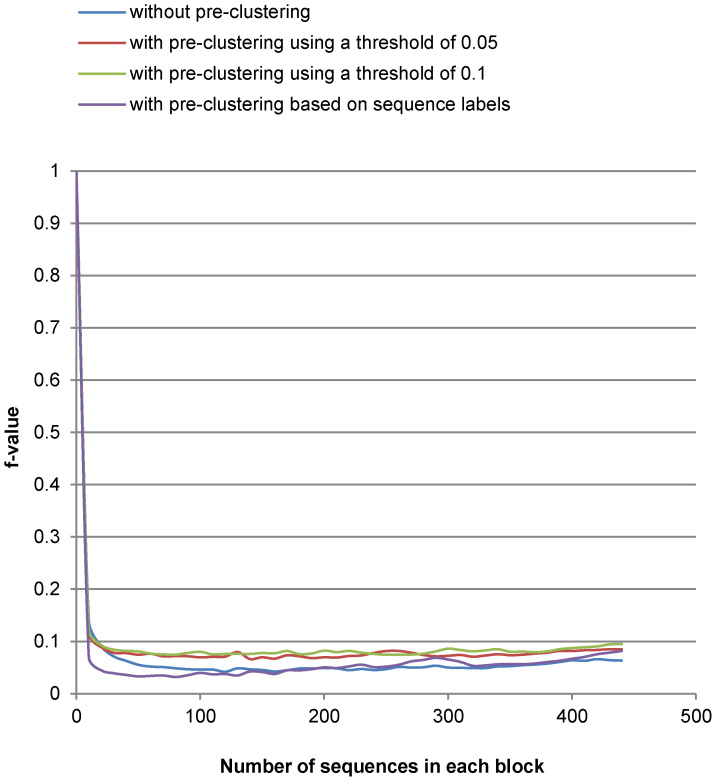
The cost (f-value) of MLC1 on Amidohydrolases protein sequences. Here the number of sequences in each block is increased by 10.

**Figure 4:**
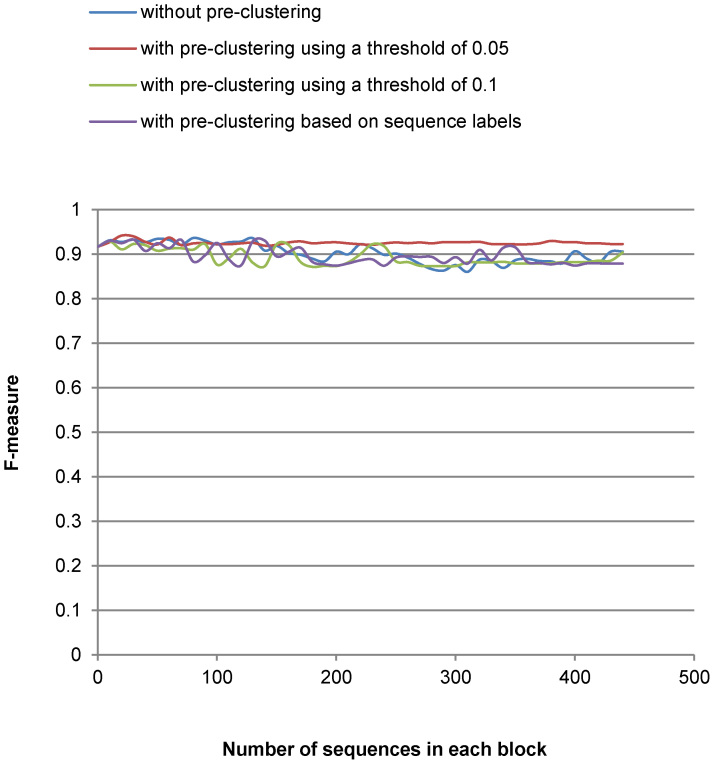
Quality (F-measure) of MLC1 clustering on Amidohydrolases protein sequences.

**Figure 5:**
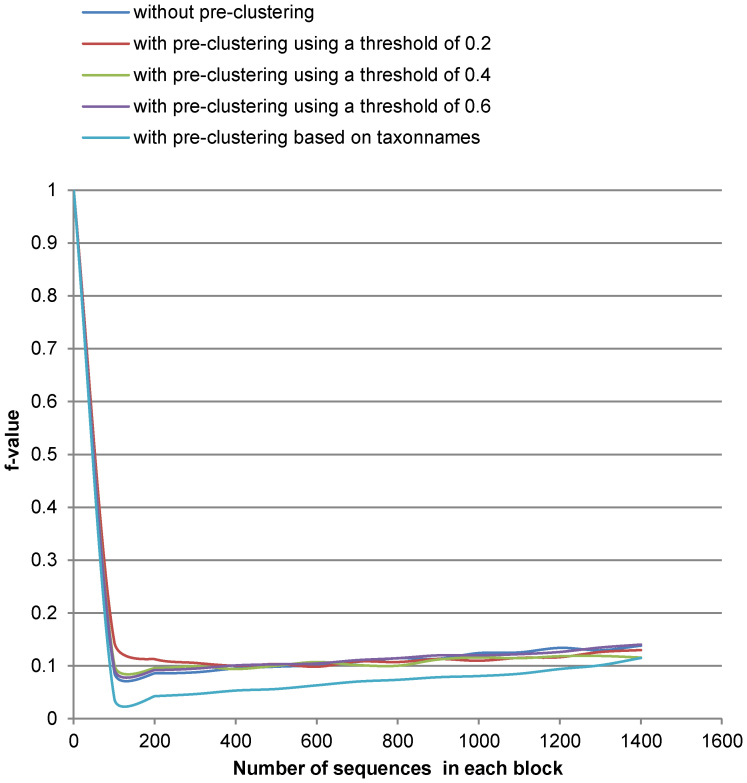
The cost (f-value) of MLC1 on the medical ITS reference sequences. Here the number of sequences in each block is increased by 100.

**Figure 6:**
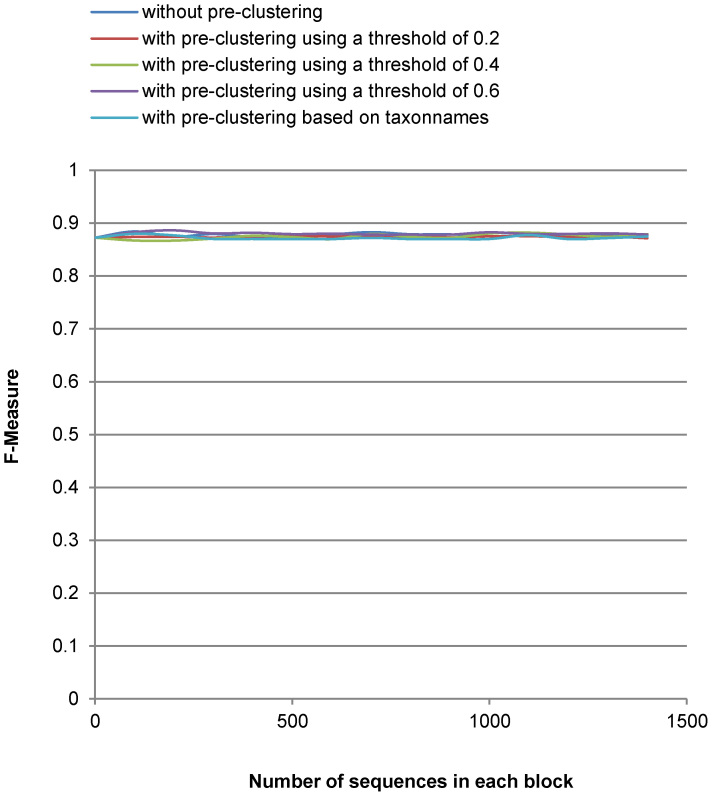
Quality (F-measure) of MLC1 clustering on the medical ITS reference sequences.

Figures 3,4,5,6 show the run-time performance and the quality of MLC1 on the two reference datasets. The lines of Figure 4 and Figure 6 are distinguished by zooming in on a portion of the axis scale in Supplementary Fig. 3–4. For the Amidohydrolases protein dataset, MLC1 was performed with the number of the sequences in each block increased by 10, and with and without pre-clustering. The best quality (*F*-measure) of MLC1 using this threshold 0.3379 was 0.941 when the dataset was first preordered by GC with the threshold 0.05 and the number of sequences in each block was 20. The *f*-value was then 0.089. The cost of MLC1 was reduced 91.1%, compared to CCBC.

For the medical fungal ITS reference sequences, MLC1 was performed with the number of the sequences in each block increased by 100, and with and without pre-clustering. The best quality (*F*-measure) of MLC1 using this threshold was 0.886. The dataset was first preordered by GC using the threshold 0.6. The next step was to cluster the dataset with MLC1 when the number of the sequences in each block was 200. The *f*-value was then 0.092. The cost of MLC1 was reduced 90.8%, compared to CCBC.

It can be seen in Figures 3,4,5,6 that the cost of MLC1 was reduced significantly, compared with CCBC whose *f*-value is 1. Furthermore, the cost and the quality of MLC1 changed slightly when the sequences were with or without pre-clustering for both datasets, and when the number of the sequences in each block was increased for the medical fungal ITS dataset. For the low-density protein dataset, the *F*-measure drops when the number of the sequences in each block was big. This is because the GC algorithm, which is shown to be very fast but not accurate with our experiments (see Table 1), was applied to group sequences in each block of MLC1. To overcome this issue, when using MLC1 to cluster low-density datasets, one could set a small number for the number of sequences in each block, or pre-cluster the sequences with a very low threshold (for example, the threshold of 0.05 for the protein dataset) to retain high accuracy. One could also apply another sophisticated algorithm such CCBC to cluster sequences in each block of MLC1 to retain high accuracy for clustering. However, the cost of MLC1 would also increase in this case.

### Run-time performance and quality of MLC

In this section, the performance of MLC is studied and compared with CCBC, TC, GC, and MLC1. To be able to produce a fixed optimal threshold for MLC1 and MLC, the number of sequences in each block of MLC1 was set as 1/10 the number of the sequences. Sequences were sorted were sorted in order of decreasing lengths initially, and no pre-clustering was applied. Furthermore, for each iteration of MLC, we set 
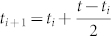
 for 1 ≤ *i* < *n*-1 and 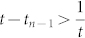. In addition, *s* = 300 with *s* the given number in Step 1 of MLC. MLC was executed with different starting thresholds on the two reference datasets.

**Figure 7:**
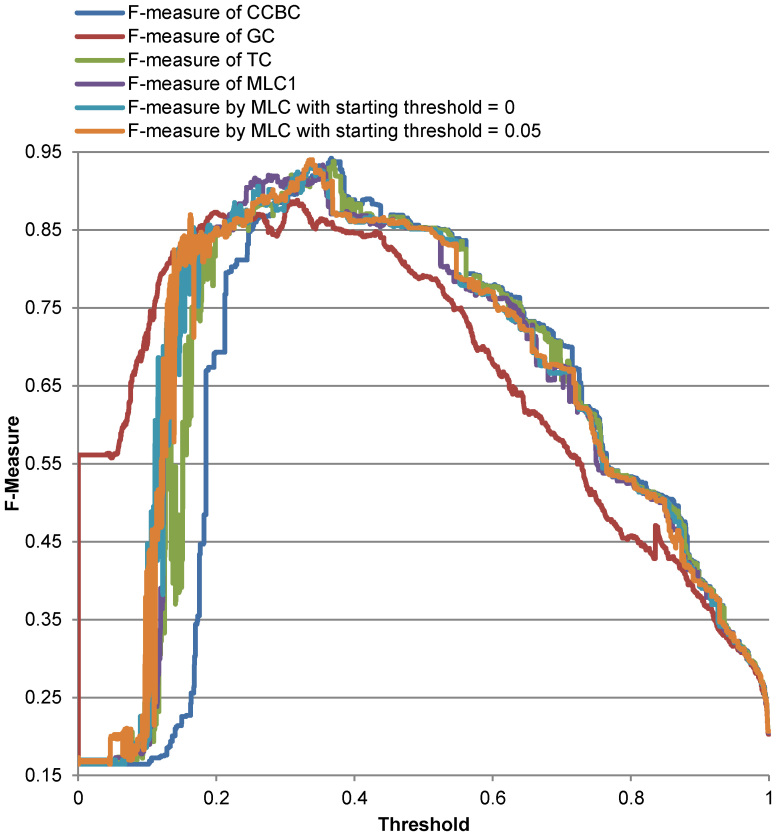
F-measure produced by CCBC, TC, GC, MLC1 and MLC on Amidohydrolases protein sequences. The threshold used for clustering is increased by steps of 0.01 ranging from 0 to 1.

**Figure 8:**
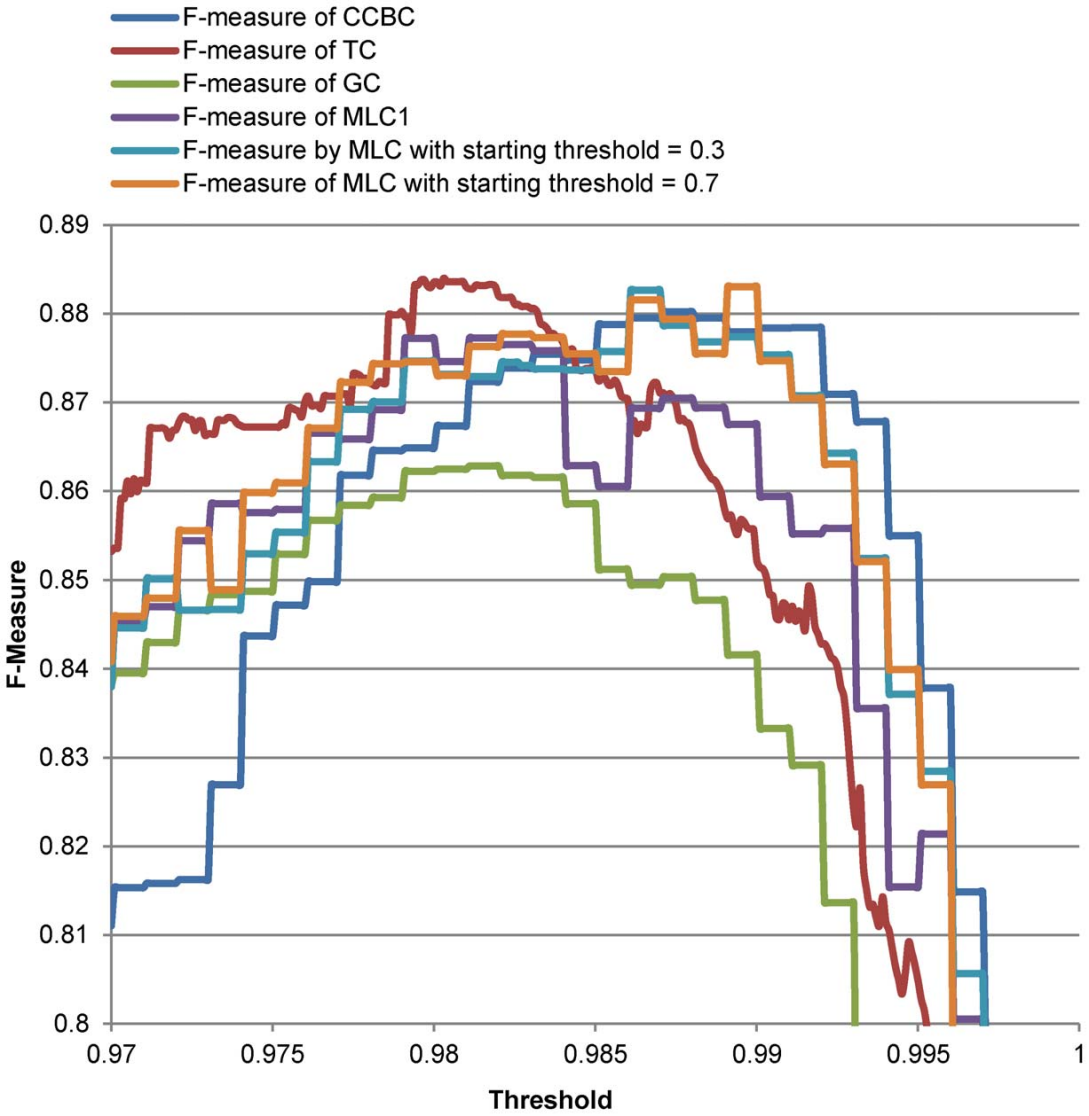
F-measure produced by CCBC, TC, GC, MLC1 and MLC on the medical fungal ITS reference sequences. The threshold used for clustering is increased by steps of 0.01 ranging from 0 to 1.

Figures 7–8 show the quality values of CCBC, TC, GC, MLC1 and MLC on the two reference datasets when the threshold used for clustering is increased by steps of 0.01 ranging from 0 to 1 for the Amidohydrolases protein dataset, from 0.97 to 1 for the yeasts, and from 0.97 to 1 for the medical fungal ITS dataset. As can be seen in these figures, except for GC, the quality of these clustering algorithms is more or less the same tools on the two reference datasets. Tables 1–2 show the best qualities (*F*-measure values) together with their corresponding optimal thresholds and the number of sequence comparisons as well as the number of the groups produced by the algorithms on the reference datasets. The number of comparisons computed for CCBC and TC is the number of comparisons needed to compute a half pair-wise similarity matrix. We note that with a right setting, MLC1 could archive a better quality for clustering. For instance, MLC1 could produce the *F*-measure of 0.9423 for the Amidohydrolases protein dataset when the number of sequences in each block was set to 20 and the sequences were pre-clustered by GC with the threshold of 0.05. For the medical fungal ITS dataset, MLC1 could produce the *F*-measure of 0.8865 when the number of sequences in each block was set to 200 and the sequences were pre-clustered by GC with the threshold of 0.6. It can be seen from the results in Tables 1–2 that MLC1 and MLC give a similar quality when clustering these datasets, compared with CCBC and TC. The number of sequence comparisons needed for MLC1 is about the same as the one of GC. However, MLC could avoid the most sequence comparisons while retaining high accuracy for clustering.

## Real-life application

Our purpose was to re-annotate all fungal ITS sequences from public databases as the ITS locus with its ease of amplification is the most widely sequenced DNA locus in fungi^25^. Furthermore, it has proven useful for identifying fungal species^26^,^27^,^28^,^29^,^30^. Recently, the ITS region has been recommended as the official fungal barcode^7^ (see also http://connect.barcodeoflife.net/group/fungi).

All 344,239 fungal ITS sequences were downloaded with the query txid4751[porgn] AND 5.8S [TITLE] from GenBank (http://www.ncbi.nlm.nih.gov) and imported into the fungal barcode database (http://www.fungalbarcoding.org) in February, 2014. Among 344,239 sequences from GenBank, there were 175,410 unidentified sequences consisting of 115,141 uncultured sequences (with names containing “uncultured” word) and 60,269 sequences with invalid names (containing a number such as “fungal sp. APA-2013”). The remaining 168,829 identified sequences were from 22,343 fungal species.

We aimed to cluster all the sequences with the optimal threshold of 0.9861 studied for the medical fungal ITS sequences on the same computer where the experiments on the two reference datasets were performed. The rMLC tool was then applied with *M* = 1000. To avoid memory constraints, maximum 1,000,000 sequence comparisons could be computed at once. The dataset was first clustered with the starting threshold of 0.95. To do this, the sequences of the dataset were first preordered based on taxon names, and then partitioned into 10 small blocks of around 30,000 sequences as given in Supplementary Table 1. Each block was clustered with the threshold 0.95 using rMLC with a starting threshold of 0.9. The number of the sequences in each block and the thresholds in each iteration of rMLC and MLC were computed as in Section Runtime-performance and quality of MLC. The number of the obtained clusters was 58,714. The run-time of this process was 7 hours and 33 minutes. All the representative sequences of the obtained cluster were then clustered into 42,219 groups in 11 hours and 5 minutes. Thus, it took 18 hours and 38 minutes to cluster the dataset with the threshold 0.95. Next, all the 42,219 groups were clustered with the optimal threshold 0.9861 in 3 hours and 5 minutes using rMLC with the starting threshold of 0.968. In total, it took 21 hours and 43 minutes to cluster all 344,239 ITS fungal sequences from GenBank with the optimal threshold 0.9861 into 89,707 groups.

To see how fast rMLC is, we also run GC on this dataset with same condition provided for rMLC. It took 242 hours and 36 minutes including the loading time to cluster the dataset into 93,249 groups by GC. It is noted that the computation of a complete similarity measure matrix alone for this dataset would take approximately 395 hours, with the assumption that there are unlimited resources.

Next, the grouping of the sequences by rMLC is studied. The number of groups of sequences before and after clustering the dataset is given in Supplementary Fig. 5. Here an unidentified sequence was considered to have its own group before clustering, while an identified sequence belonged to a group labeled with its species name. After clustering, there were 32,292 groups of identified sequences and 57,415 groups of unidentified sequences. Based on the result obtained by rMLC, there were 5272 uncultured and 13,874 sequences with invalid names being identified. In particular, these sequences were partitioned into 2649 groups labeled with a species name. The remaining unidentified sequences were clustered into the 57,415 groups mentioned above. Furthermore, there were 2389 species being merged into groups of other species. This means that these species could not be distinguished by ITS region with the threshold 0.9861. Finally, there were 5067 species being split into 16,868 groups since the minimum similarity measure in these species was lower than 0.9861.

We cannot guarantee that the clusters created by rMLC with the threshold 0.9861 indeed are consistent with the sequence family as the distribution of the species is not equal in nature. However, with our approach, we hope to speed up the validation of the sequences and help to find out quickly considerable portion of potentially undescribed diversity.

## Conclusions and future work

In this paper, the multilevel clustering algorithms MLC1, MLC and rMLC were proposed to avoid a major number of sequence comparisons while retaining high accuracy for sequence clustering. MLC1 was created based on the connected components based clustering (CCBC) and the greedy clustering (GC) algorithms, while MLC was developed based on MLC1 to cluster large-scale datasets with number of the obtained group expected to be big. The rMLC tool was developed based on MLC to further improve the speed and accuracy of clustering.

The advantage of these algorithms is that they can benefit the accuracy of current sophisticated data-partitioning methods such as CCBC and TC as well as the efficiency of the most efficient tool in clustering such as GC. With MLC and rMLC, we could also avoid memory problems when dealing with large-scale datasets. We have shown by experiments that while the speed of MLC1 and MLC is comparable with GC, there is no or only a marginal drop in accuracy when compared them with CCBC and TC. By applying rMLC, we were able to cluster 344,239 ITS sequences from Genbank at species level within 22 hours while and it took 242 hours and 36 minutes for the greedy clustering method on the same computer. In addition, it would take 395 hours only to compute a matrix of similarity measures alone for traditional clustering methods (with the assumption that there is no memory-constraint).

It is not straightforward for the users to provide a right input setting for MLC algorithms to cluster a dataset in order to archive the best quality of clustering while being effective. MLC1 is simple to use. But, it is not applicable to cluster large-scale datasets with a big number of expected groups. MLC and rMLC require knowledge about the dataset such as the density of the sequences to give a right input set of increasing thresholds for clustering. However, this input set can be learnt by studying a portion of the dataset.

The MLC algorithms have been applied to automatically re-annotate and curate the entire barcode reference database (currently *ca.* 80k) at the CBS-KNAW fungal collection (http://www.cbs.knaw.nl). This helps to find out a considerable portion of potentially undescribed diversity, and to speed up the whole process of sequence validation which is the most severe bottleneck in the barcoding project at our institute^31^. A tool based on MLC has been developed and implemented to support MycoBank (http://www.mycobank.org) for *online* identification of fungal species. In the future, we will introduce parallelism to MLC as it can easily be made cloud-enabled. This could significantly speed up the whole process further and avoid memory problems when dealing with large-scale datasets.

Although in this work, MLC and its variants have been developed for taxonomic purposes, they are clearly applicable to the management of the large amounts of data in other research fields. For instance, it can be applied in the processing of gene expression data, in protein identification, in spectrometric analysis, and in metabolomics.

## Author Contributions

D.V. developed and implemented the algorithms, and wrote the manuscript. S.S. and V.R. developed and implemented the DNA comparison function. C.W. compared MLC with Transitivity Clustering using the protein dataset. G.C. proposed the method to calculate the representative sequence of a group of MLC. R.R. contributed to the calculation of the complexity of the algorithms, read and commented on the manuscript. V.R. and J.B. contributed to the algorithms, supervised the research and revised the manuscript. All authors reviewed the manuscript.

## Supplementary Material

Supplementary Information Supplementary Information

## Figures and Tables

**Table 1:** Results of clustering on Amidohydrolases protein sequences

	Optimal threshold	Best F-measure	Number of comparisons	Number of groups
**CCBC**	0.3661	0.9421	374545	95
**TC**	0.311	0.9281	374545	91
**GC**	0.3167	0.8882	21593	99
**MLC1**	0.3538	0.9338	34596	97
**MLC (*t*_1_ = 0)**	0.3387	0.9359	13056	91
**MLC (*t*_1_ = 0.005)**	0.3357	0.9402	16666	93

**Table 2:** Results of clustering on the medical fungal ITS sequences

	Optimal threshold	Best F-measure	Number of comparisons	Number of groups
**CCBC**	0.9871	0.8802	3918600	452
**TC**	0.9803	0.884	3918600	449
**GC**	0.9811	0.8628	492781	480
**MLC1**	0.9791	0.8772	359831	398
**MLC (*t*_1_ = 0.3)**	0.9861	0.8826	142531	463
**MLC (*t*_1_ = 0.7)**	0.9891	0.883	152224	509
