# Supplementary material for: Massive fungal biodiversity data re-annotation with multi-level clustering
Source: Sci Rep. 2014 Oct 30;4:6837. doi: 10.1038/srep06837 (PMC4213798; doi:10.1038/srep06837)
Supplement: Supplementary Information [file srep06837-s1.pdf]

Massive fungal biodiversity data re-annotation with multi-level clustering

Duong Vu[1], Szániszló Szoke[1], Christian Wiwie[2], Jan Baumbach[3], Gianluigi Cardinali[4], Richard Röttger[2,3], Vincent Robert[1]
[1] Bioinformatics group, CBS-KNAW Fungal Biodiversity Centre, Utrecht, The Netherlands
[2]Computational Systems Biology, Max Planck Institute for Informatics, Saarbrucken, Germany
[3]Institute for Mathematics and Computer Science, University of Southern Denmark, Odense, Denmark
[4]University of Perugia, Perugia, Italy

Supplementary Figure 1: F-Measures produced by MLC1 on Amidohydrolases protein sequences when the number of the sequences in each block is 200. The sequences were sorted in order of decreasing lengths. The threshold used for clustering is increased by steps of 0.01 ranging from 0 to 1. The best F-measures of MLC1 on this dataset when the representative sequences were chosen as the first sequence, the central sequence and the almost-central sequence are 0.9188, 0.93 and 0.942 respectively. It is noted that the almost-central sequence of a group was computed with *k*=10.

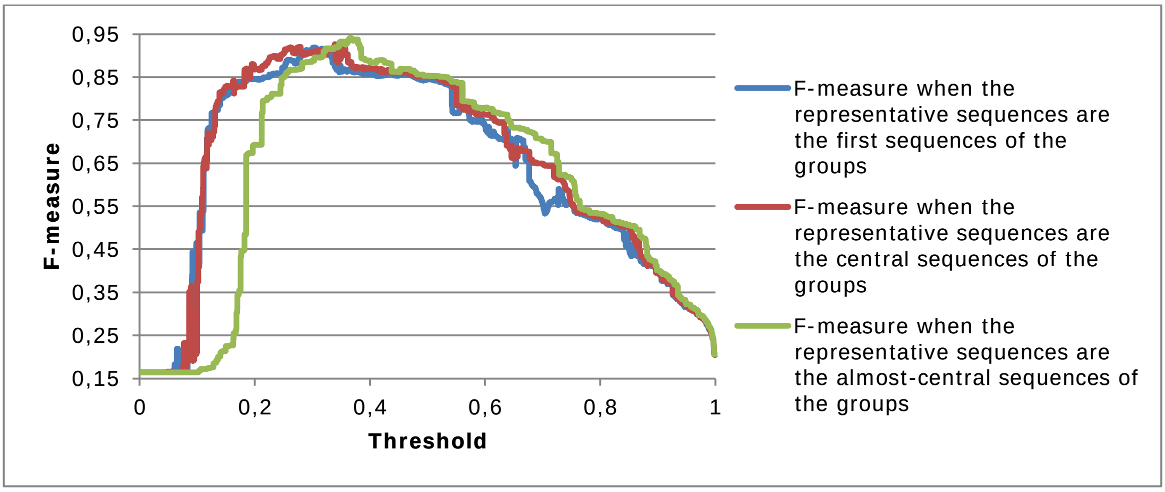

Supplementary Figure 2: F-Measures produced by MLC1 on medical fungal ITS sequences when the number of the sequences in each block is 200. The sequences were sorted in order of decreasing lengths. The threshold used for clustering is increased by steps of 0.01 ranging from 0.97 to 1. The best F-Measures of MLC1 on this dataset when the representative sequences were chosen as the first sequence, the central sequence and the almost-central sequence are 0.8666, 0.8753 and 0.8753 respectively. It is noted that the almost-central sequence of a group was computed with *k*=10.

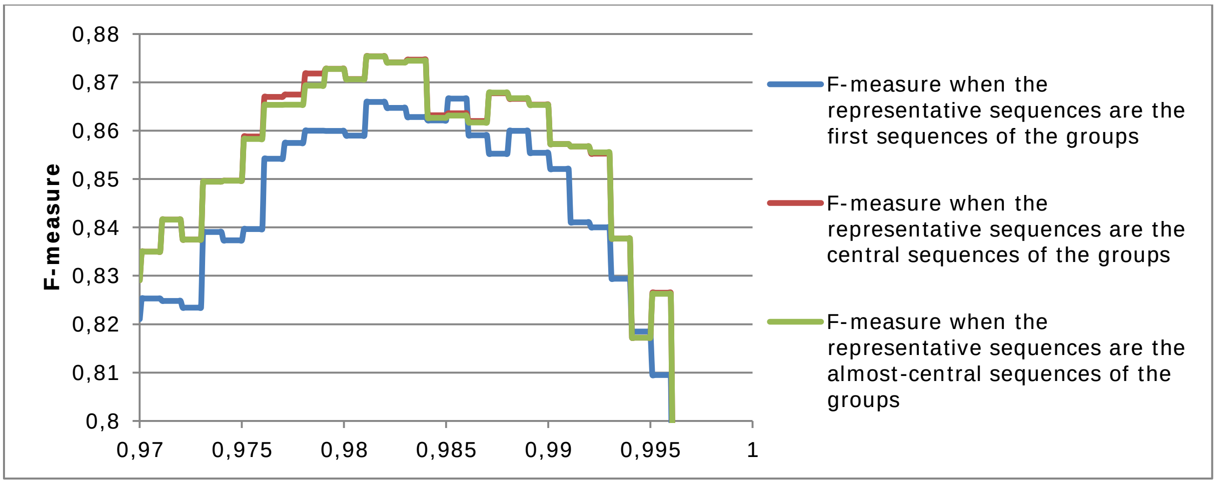

Supplementary Figure 3: Zooming in the quality (*F*-measure) of MLC1 clustering on Amidohydrolases protein sequences.

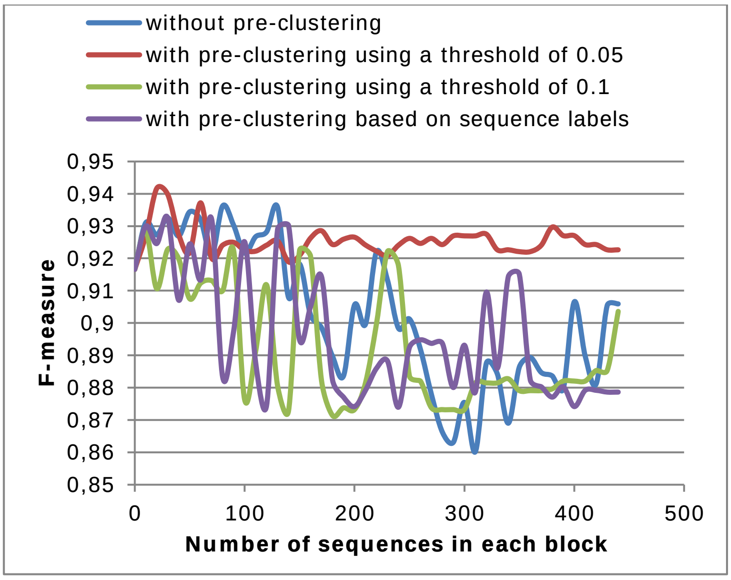

Supplementary Figure 4: Zooming in the quality (*F*-measure) of MLC1 clustering on the medical ITS reference sequences.

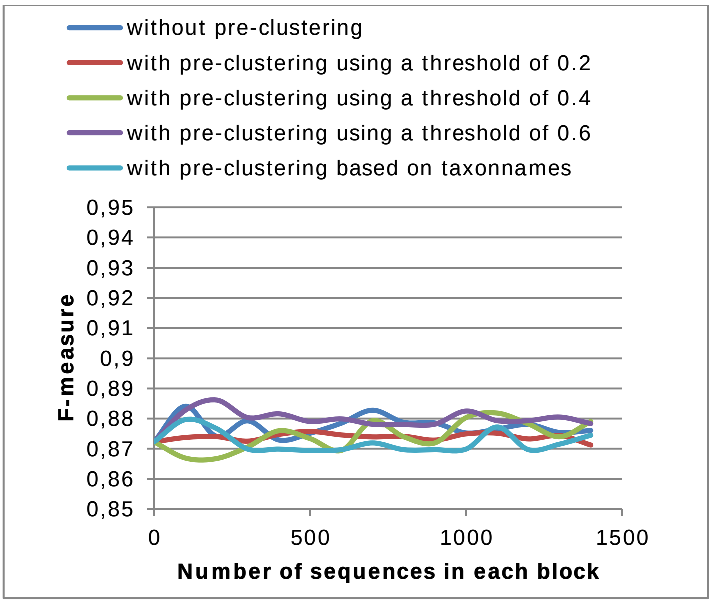

Supplementary Figure 5: Number of groups before and after clustering.

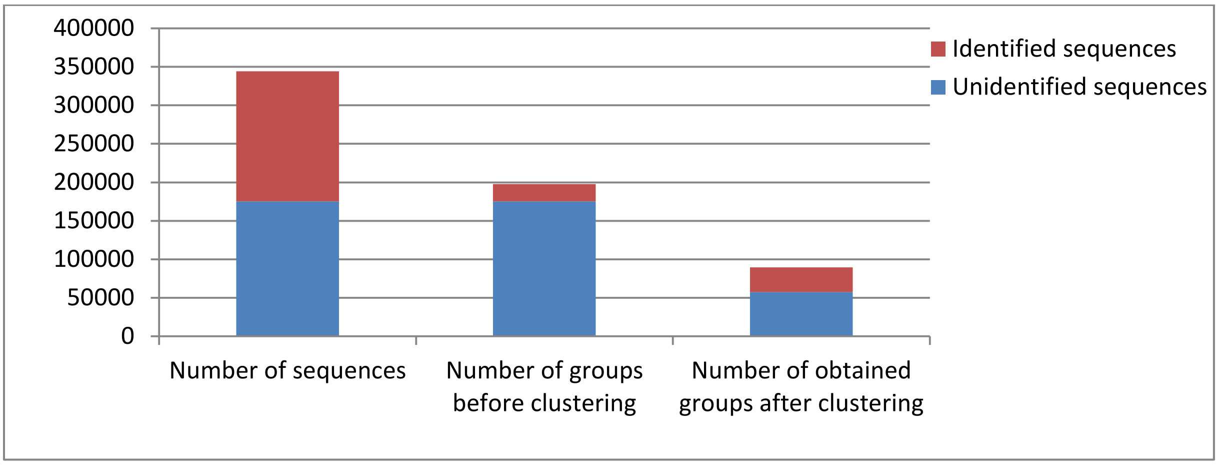

Supplementary Table 1: Results of rMLC with the threshold 0.95 where *n* is the number of sequences in each block.

	B1	B2	B3	B4	B5	B6	B7	B8	B9	B10
n	26,997	28,78	24,728	14,310	25,241	28,603	21,791	29,702	88,703	55,383
Time	23m	26m	20m	33m	30m	20m	21m	22m	2h15m	2h3m
